# Ecological Energetics of an Abundant Aerial Insectivore, the Purple Martin

**DOI:** 10.1371/journal.pone.0076616

**Published:** 2013-09-25

**Authors:** Jeffrey F. Kelly, Eli S. Bridge, Winifred F. Frick, Phillip B. Chilson

**Affiliations:** 1 Oklahoma Biological Survey, University of Oklahoma, Norman, Oklahoma, United States of America; 2 Department of Biology, University of Oklahoma, Norman, Oklahoma, United States of America; 3 Ecology and Evolutionary Biology Program, University of Oklahoma, Norman, Oklahoma, United States of America; 4 Ecology and Evolutionary Biology, University of California Santa Cruz, Santa Cruz, California, United States of America; 5 School of Meteorology and Advanced Radar Research Center, University of Oklahoma, Norman, Oklahoma, United States of America; University of Regina, Canada

## Abstract

The atmospheric boundary layer and lower free atmosphere, or aerosphere, is increasingly important for human transportation, communication, environmental monitoring, and energy production. The impacts of anthropogenic encroachment into aerial habitats are not well understood. Insectivorous birds and bats are inherently valuable components of biodiversity and play an integral role in aerial trophic dynamics. Many of these insectivores are experiencing range-wide population declines. As a first step toward gaging the potential impacts of these declines on the aerosphere’s trophic system, estimates of the biomass and energy consumed by aerial insectivores are needed. We developed a suite of energetics models for one of the largest and most common avian aerial insectivores in North America, the Purple Martin ( *Progne* *subis* ). The base model estimated that Purple Martins consumed 412 (± 104) billion insects*y^-1^ with a biomass of 115,860 (± 29,192) metric tonnes*y^-1^. During the breeding season Purple Martins consume 10.3 (+ 3.0) kg of prey biomass per km^3^ of aerial habitat, equal to about 36,000 individual insects*km^-3^. Based on these calculations, the cumulative seasonal consumption of insects*km^-3^ is greater in North America during the breeding season than during other phases of the annual cycle, however the maximum daily insect consumption*km^-3^ occurs during fall migration. This analysis provides the first range-wide quantitative estimate of the magnitude of the trophic impact of this large and common aerial insectivore. Future studies could use a similar modeling approach to estimate impacts of the entire guild of aerial insectivores at a variety of temporal and spatial scales. These analyses would inform our understanding of the impact of population declines among aerial insectivores on the aerosphere’s trophic dynamics.

## Introduction

With increasing human use of the atmospheric boundary layer and lower free atmosphere (aerosphere) for energy, communication, transportation, and remote sensing, our need to understand the aeroecology of animals whose life histories depend on this environment has also increased [1]. Understanding the dominant ecological processes occurring in the aerosphere, including consumption of insects by vertebrate predators, bolsters our basic understanding of trophic interactions, which is important for effective conservation and management of aerial species and their habitats (e.g., [2,3]). These trophic relationships are of added interest owing to regional population declines in avian insectivores across broad spatial scales [4,5]. Although causes of these population declines remain unclear [4,6], when they are coupled with increasing human activity in the aerosphere, they generate conservation concern [7].

It is difficult to quantify the cumulative trophic impact of insectivorous birds because, at a minimum, it requires estimates of (1) total numbers of these birds, (2) energetic requirements of those birds, and (3) energy content of insect prey. Despite this difficulty, a quantitative model of the magnitude of energy flowing through aerial trophic systems could provide a starting point for investigations of the potential ecological consequences of changes in the abundance of both predators and prey [8]. These types of estimates are relatively rare in the ecological literature [9].

One of the most abundant and widely distributed aerial insectivores in North America is the Purple Martin ( *Progne* *subis* ) [10]. Similar to other aerial insectivores, Purple Martin populations in the northern part of the United States and in Canada have declined over the past 20 years [5]. Unlike most other swallows, Purple Martins breed primarily in structures provided by humans. This synanthropic life-history adds an additional level of complexity to Purple Martin population dynamics [11]. In particular, changes in human demography can alter availability of nesting habitats. However, the Purple Martin’s close association with humans has also resulted in a trove of high-quality demographic data, which are critical to estimating their ecological energetics [12].

We used existing information on demography and energetics of Purple Martins to create a mathematical model of population and energetic dynamics of the species’ global population through their annual cycle. The model estimates the number of birds alive on each day of the year and both the number and biomass of insects consumed daily by Purple Martins. Our objective was to estimate the magnitude of the ecological energy flow attributable to Purple Martins as one representative aerial insectivore.

## Methods

### Purple Martin Population Model

Based on the North American Breeding Bird Survey, the North American Landbird Conservation Plan [13] estimated that the global abundance of Purple Martins is 10 million individuals. Rich et al. [13] provide confidence intervals around this estimate of +/- 50%, suggesting that the true number of Purple Martins is likely to be between 5 and 15 million birds. This estimate reflects the abundance of adult birds because it is derived from breeding bird surveys conducted early in the breeding season.

By mid-summer the total number of Purple Martins in North America includes adults plus their young-of-the-year. Tarof and Brown [10] reviewed estimates of offspring production per breeding adult and reported a range of 3 to 4.4 young per nest, except during a cold, wet year when production was only 0.3 young per nest [14]. Several studies reported that most adults of both sexes breed each year [15] with the highest reported percentage of non-breeding floaters being 13% of males [16]. There are also reports of females as non-breeding floaters, although the percentage is low (0.6% [10]).

**Figure 1:**
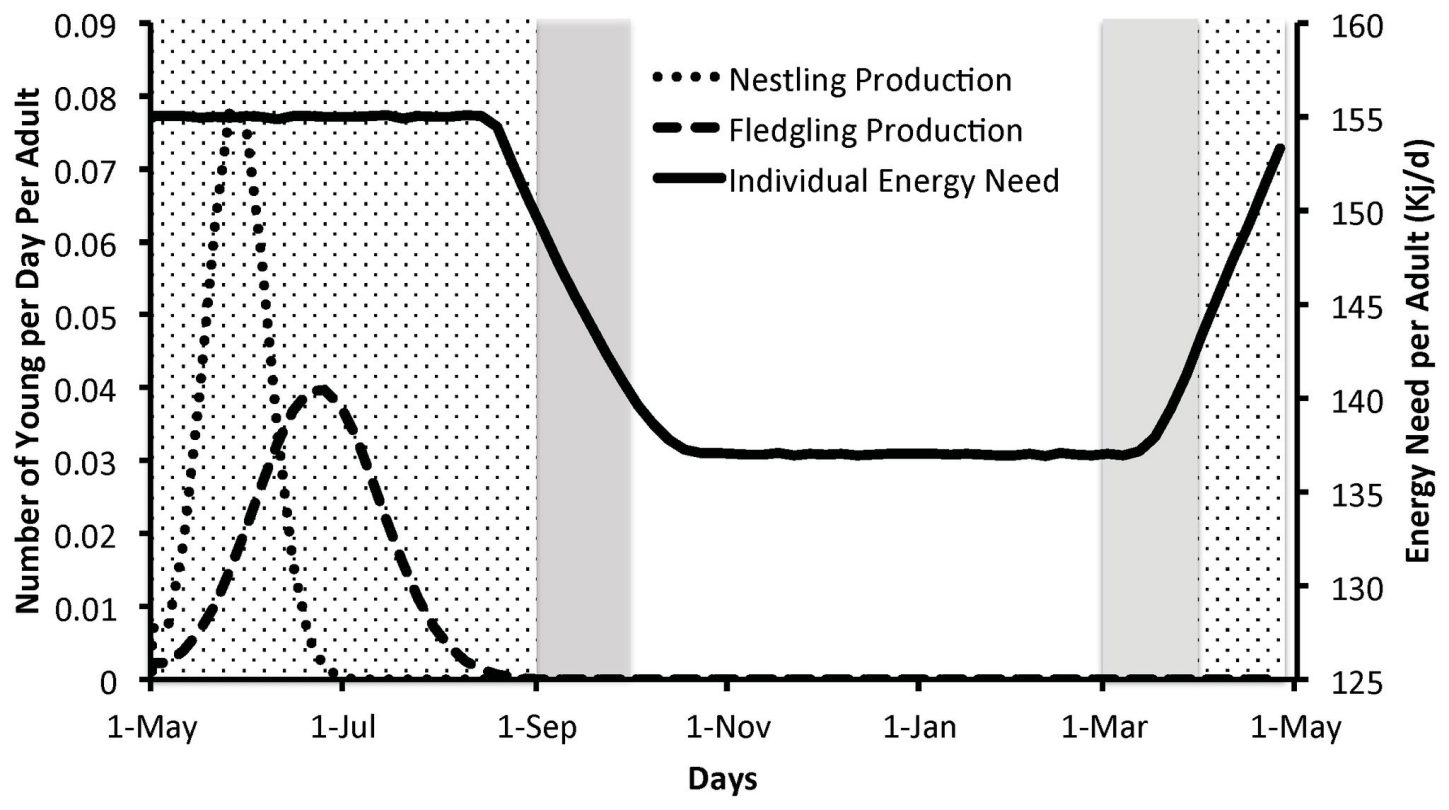
Phenology of energy need of adult Purple Martins (solid line), nestling production per adult (dotted line), and fledgling production per adult (dashed line) across one year. Background shading indicates phase of the annual cycle as breeding season (stippling), migration (gray), and winter (no shading).

Based on results of these studies, we made the simplifying assumption that all adult Purple Martins breed each year and produce 2 young. We assumed a 1:1 sex ratio. In the model, Purple Martins begin nesting on 1 May (day 1 of the model) with most clutches being completed on 12 May. Based on this timing, the average hatch date is 28 May (SD = 10 d), which follows a 16-day incubation period. Nestlings fledge and become independent, on average, 26 d later on 23 June (SD = 20 d). This timing of the nesting stages is typical of Purple Martins throughout much of their range [10]. We modeled the timing of nestling and fledgling production using a normal probability distribution function through the breeding season (Figure 1).

Stutchbury et al. [12] estimated annual survival rates of one-year-old, two-to-four-year-old, and older female Purple Martins to be 0.48, 0.62, and 0.51, respectively, or 0.58 across all adult age groups. We estimated daily survival of adult Purple Martins to be 0.9985, which yields an annual survival rate of 0.58. Even though there are clearly age-based differences in reproduction and survival of adult Purple Martins, estimating parameters for each adult age and sex class of Purple Martins was unnecessary for the purpose of estimating energy flow and inclusion of these extra model parameters would unnecessarily increase error propagation in the model [17].

Survival of hatch-year birds during their 1^st^ year is estimated to be 0.27 in Purple Martins [18]. We used a daily survival probability of 0.9964 for hatch-year birds (i.e., young-of-the year), which yields an annual survival rate of 0.27. A model using demographic parameters drawn directly from the literature (adult survival = 0.58*y^-1^, 2 young * adult^-1^, and juvenile survival = 0.27*y^-1^), resulted in an annual population growth rate (λ) of about 17% (λ = 1.17). This growth rate is considerably higher than is sustainable or expected based on empirical data [5]. We suspect that the primary difference between empirical evidence for recent population declines and our base model population growth of 17% per year is due to elevated reproductive output among Purple Martins that are the subject of intensive studies reported in the literature relative to the average Purple Martin. The quality and maintenance of housing by people can strongly impact the reproductive output of birds and we suspect that the housing and protection of martins that are the subject of intensive study is likely to be better than average.

Rather than use this model with an unrealistically high growth rate as the basis for all of our comparisons, we chose to compare among models with minimal annual population change (λ =1). We argue that these models are optimal for comparisons of energy dynamics because (1) the long-term average λ for Purple Martin populations must be close to 1 and (2) based on breeding bird surveys, this value is within 1% of the value for recent Purple Martin population dynamics, which indicate an average decline over the past few decades of about 1% per year range wide (λ =0.99). Therefore, models with λ=1 have the diagnostic advantage of being both currently and historically relevant when compared to models that estimate unsustainable population increases or declines. To achieve a population growth rate with a long-term average λ of 1.0, we generated three additional models in which we reduced either adult survival (0.43*y^-1^ = 0.9977*d^-1^), production of young (1.45*adult^-1^), or juvenile survival (0.19*y^-1^ = 0.9954*d^-1^). In each of these three models the reduced parameter value resulted in a stable population.

To model the estimated variance in total number of adult Purple Martins, we performed 10,000 model simulations, which were initialized with a number of adult Purple Martins chosen at random from a normal distribution with a mean of 10 million individuals and a standard deviation of 2.5 million (Table 1). We chose a standard deviation of 2.5 million Purple Martins because 2 standard deviations approximates the confidence interval of +/- 5 million Purple Martins published by Rich et al. [13]. To understand how sensitive model outputs were to choice of initial population size we also ran 10,000 simulations with mean initial abundances of 7.5 and 12.5 million Purple Martins.

**Table 1:** Parameters used in a model to estimate abundance and energy consumption of Purple Martins. Values are reported as means with standard deviations. Values for each of 10,000 simulations were drawn from normal distributions.

Parameter	Mean Value (SD)	Units	Source
Demography			
Abundance	1x10^7^ (2.5x10^6^)	Individuals	[13]
Adult Survival Rate	0.9985 (0.001)	d^-1^	[12]
Juvenile Survival Rate	0.9964 (0.001)	d^-1^	[18]
Total Offspring Number	2.0	Adult^-1^	[10]
Energetics			
Energy Need	137 or 155 (1.37 or 1.55)	kJ*d^-1^	[19]
Prey Energy	23 (2.3)	kJ*g^-1^	[24]
Prey Size	20 (2.0)	mm	[21]
Prey Dry Mass	-7.761+(0.34975*Prey Size)-0.0039315*Prey Size^2^	ln (g)	[25]
Prey Wet Mass	-6.972+(0.3687*Prey Size)-0.0041725*Prey Size^2^	ln (g)	[25]

### Purple Martin Energetics

Utter and Lefebvre [19] reported that daily energy expenditures of breeding Purple Martins range from about 137 kJ*d^-1^ for males to about 175 kJ*d^-1^ for females engaged in feeding nestlings. This difference in energy expenditure is due to females feeding young whereas the males engaged in little parental care. This situation is likely atypical, because males generally provision offspring as frequently as females [20]. Therefore, to estimate the energy expenditure of single independent Purple Martins, we assumed the values for males reported by Utter and Lefebvre [19] were representative of non-breeding birds. To represent the daily energy requirement of a single non-breeding Purple Martin, we used a randomly drawn value from a normal distribution with mean 137 kJ*d^-1^ (SD = 1.37kJ* d^-1^; 1% of the mean). For breeding Purple Martins we assumed that energy expenditure was 155 kJ*d^-1^(SD =1.55kJ* d^-1^), that is, intermediate between values for breeding males and females [19]. We used an intermediate value for breeding birds because both parents typically engage in parental care, unlike those birds measured by Utter and Lefebvre [19]. Our model assumed that Purple Martins require this 155 kJ*d^-1^ from mid March through mid August, which roughly corresponds to the breeding season (Figure 1). While the duration of the high-energy period may overestimate the length of the breeding period for any particular Purple Martin, the difference is likely offset by other energetically expensive activities (molt and migration) that occur in this time frame but that are not explicitly accounted for in our model.

Purple Martin diets contain many taxonomic orders of insects [21,22,23]; as well as a few non-insect prey. These insects all contain different amounts of energy*g^-1^. Brooks et al. [24] tested whether using a mean value for the energy content of insect prey was adequate to explain the ecological energetics of insectivorous vertebrates. They concluded that a constant value of 23J*mg^-1^ (=23 kJ*g^-1^ of dry mass) was adequate for studies of generalist vertebrate insect predators. Based on this result, we assumed insects consumed by Purple Martins provide 23 kJ*g^-1^ (SD = 0.23 kJ) of energy, meaning that a non-breeding Purple Martin needs to eat about 6.0 g of dry mass of insects*d^-1^ to meet basic energy requirements (137 kJ*d^-1^) and that a breeding bird needs about 6.7 g*d^-1^ to reap 155 kJ*d^-1^.

To convert from dry mass to numbers of insects consumed we used the relationships in Sage [25]. We use Sage’s [25] dry mass vs. length relationship (R^2^ = 0.86) and wet mass vs. length (R^2^ = 0.87) for Insecta to estimate the number of insects that Purple Martins would need to eat to meet average daily energetic requirements. The relationships were based on data for 153 adult insects from Orthoptera (n =36), Hemiptera (n = 26), Coleoptera (n = 29), Lepidoptera (n = 25), Diptera and Hymenoptera (n = 37). We estimated the mass of insects by drawing at random a mean insect length from a normal distribution with an average of 20 mm (± 2mm). The energy provided by insects of this length was estimated from the equations of Sage [25]. We modeled all of the Purple Martin abundance and energetics relationships using a Matlab script ( [26]; Table 2).

**Table 2:** Psuedocode describing the logic and structure of the Matlab script used to model abundance and energetics of Purple Martins during one year.

ASSIGN PDF (probability distribution function) for proportion of fledglings that fledge on a given day
ASSIGN PDF for proportion of nestlings in a nest on a given day
FOR the specified number of iterations
SET the initial number of adult birds based on a PDF
FOR every day of the year (starting on May 1)
SET the adult survival rate based on a PDF
SET the juvenile survival rate based on a PDF
SET the insect energy content by mass based on a PDF
SET the insect size based on a PDF
SET the daily energy need of breeding birds based on a PDF
SET the daily energy need of non-breeding birds based on a PDF
SET the dry insect mass according to Sage [25]
SET the wet insect mass according to Sage [25]
IF the day is between May 1 and August 17 inclusive
CALCULATE the new number of birds assuming adults and fledglings
CALCULATE the energy needed per day per bird taking breeding into account
ELSE
CALCULATE the new number of birds assuming adults only
CALCULATE the energy needed per day per bird (no breeding)
ENDELSE
CALCULATE the total bird abundance
CALCULATE the number of insects consumed per bird per day
CALCULATE the number of insects consumed by all birds in a day
CALCULATE the dry mass of insects consumed by all birds in a day
CALCULATE the wet mass of insects consumed by all birds in a day
ENDFOR
ENDFOR
CALCULATE the mean total bird abundance for all iterations
CALCULATE the standard deviation of total bird abundance for all iterations
CALCULATE the mean total insects consumed per day for all iterations
CALCULATE the standard deviation of total insects consumed per day for all iterations
CALCULATE the mean total biomass consumed per day for all iterations
CALCULATE the standard deviation of total biomass consumed per day for all iterations

Parameters values are listed in Table 1 or in the Methods. The script can be obtained by request.

To estimate the number and biomass of insects consumed by Purple martins in different phases of the annual cycle we divided the model estimates for biomass and number of insects consumed into a breeding period (1 April to 31 August), fall migration (1 to 30 September), winter residency (1 October to 28 February), and spring migration (1 to 31 March). We used an existing breeding range map [27] to estimate the land area within the range of the Purple Martin during these 4 phases of the annual cycle. We estimated that the Purple Martin population was spread over 5.72 million km^2^, 2.97 million km^2^, and 11.6 million km^2^ of land area in the breeding, migratory, and winter periods. We made the simplifying assumption that Purple Martins foraged in the lowest 1km of the aerosphere and converted km^2^ of land area to km^3^ of habitat volume in which the birds forage. We calculated the biomass and number of insects consumed by Purple Martins by dividing the model estimates of number and biomass of insects consumed by estimates of habitat volume for each phase of the annual cycle.

## Results

**Figure 2:**
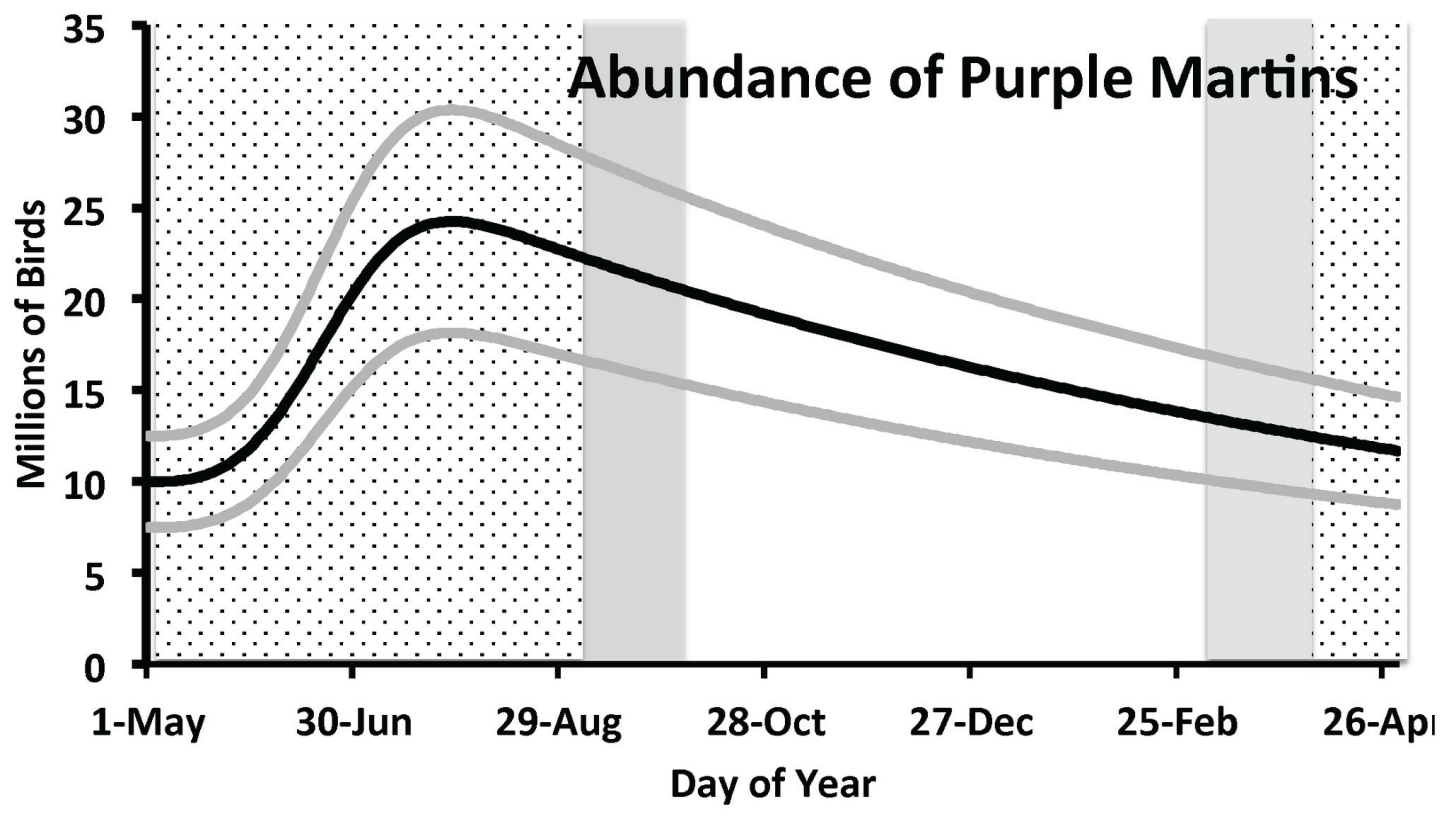
Estimated mean number (black line) ± standard deviation (gray lines) of Purple Martins based on 10,000 replications of a demographic simulation model. Background shading indicates phase of the annual cycle as breeding season (stippling), migration (gray), and winter (no shading).

**Figure 3:**
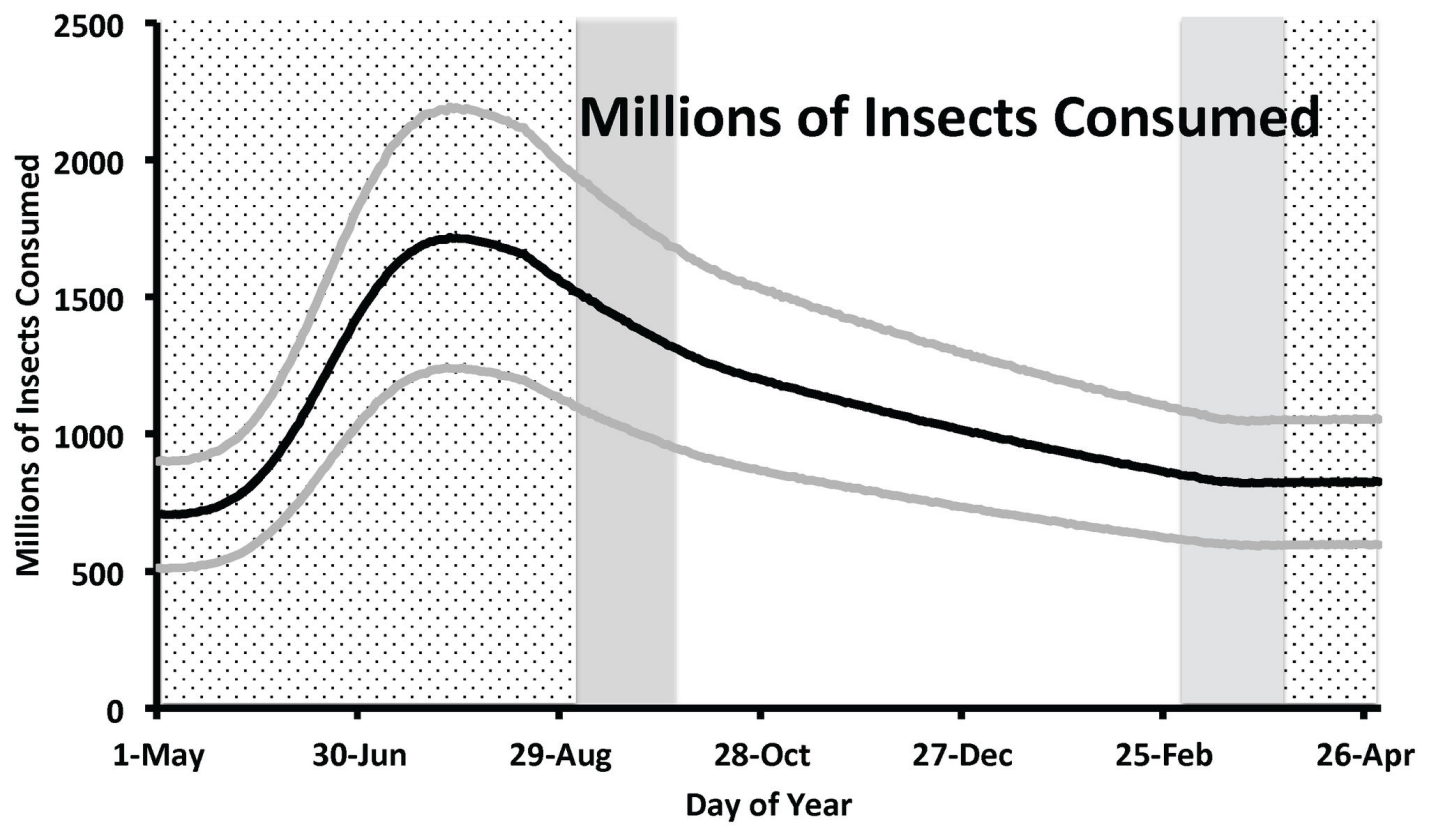
Estimated mean number (black line) ± standard deviation (gray lines) of insects consumed by Purple Martins based on 10,000 replications of an energetics simulation model. Background shading indicates phase of the annual cycle as breeding season (stippling), migration (gray), and winter (no shading).

**Figure 4:**
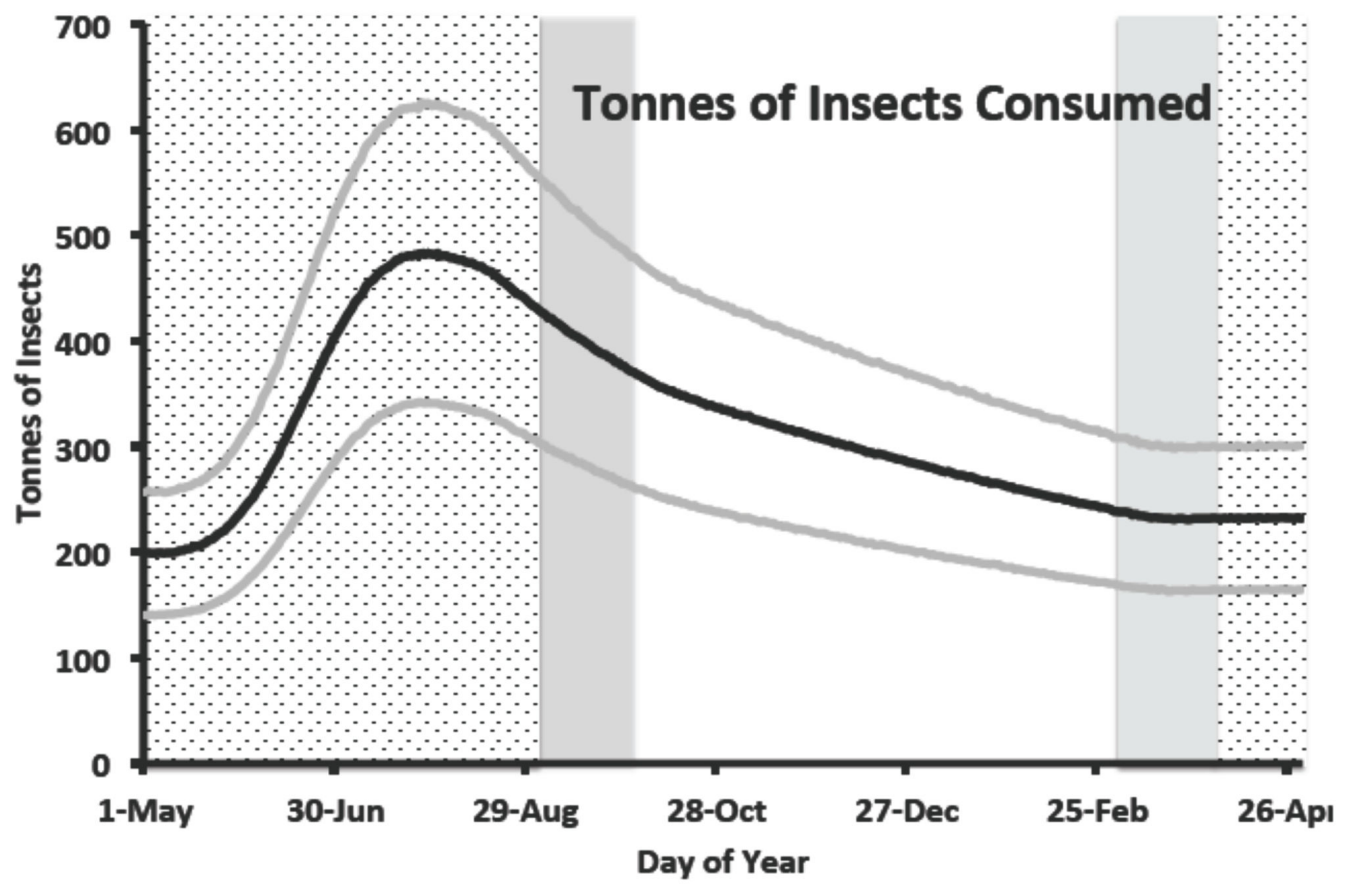
Estimated mean mass (in tonnes, black line) ± standard deviation (gray lines) of insects consumed by Purple Martins based on 10,000 replications of an energetics simulation model. Background shading indicates phase of the annual cycle as breeding season (stippling), migration (gray), and winter (no shading).

The average starting population of Purple Martins in our base model was 9,986,700 ± 2,511,900 Purple Martins on 1 May. On the last day of the model (30 April; one year later) the mean population was 11,682,800 ± 2,944,400 Purple Martins for an average annual λ = 1.17. The population size peaked on 29 July at an average of 24,242,600 ± 6,102,400 Purple Martins (Figure 2). These Purple Martins consumed a total of 412 (± 104) billion insects in a year with a maximum daily consumption of 1.7 (±0.5) billion insects (Figure 3; Table 3). The mass of these insects summed to an annual total biomass of 115,860 (± 29,192) metric tonnes. The maximum average daily consumption was 484 (± 142) tonnes of insect biomass on 28 July (Figure 4). Relative to the base model, these estimates decline by 8-15% in three models with demographic parameters that were adjusted downward so that annual population growth was < 1% (Table 3). All else being equal, biomass of insects consumed annually was linearly related to the initial abundance of Purple Martins (Table 3).

**Table 3:** Projected number (billions) and biomass (tonnes) of insects consumed by Purple Martin populations annually.

Number of Martins			Parameter Reduced so that Lambda = 1		
	Output Variable	Literature Model	Adult Survival	Juvenile Survival	Young
7.5 Million	Insects consumed	307	287	289	262
	Biomass consumed	86,638	80,771	81,316	73,742
**10 Million**	**Insects consumed**	**412**	383	386	348
	**Biomass consumed**	**115,860**	107,770	108,520	97,911
12.5 Million	Insects consumed	515	478	482	438
	Biomass consumed	144,980	134,480	135,700	123,330

Projections are based on estimated abundance and demographic rates from the literature (base model). This model results in an improbably high annual growth rate (lambda = 1.17). Outputs of models with negligible population change are also presented. These models differed from the base model by having decreased daily adult survival rate (reduced to 0.9977), juvenile survival (reduced to 0.9954), or young per adult (reduced to 1.45). Outputs are the means of 10,000 simulations. Results of the model based most closely on the literature (base model) are in bold.

We estimated that Purple Martins consume more cumulative biomass and individual insects during the summer breeding period in the USA and Canada than at other phases of the annual cycle (Table 4). This result stems from the birds being in the breeding range for 5 months, having relatively high energetic requirements during that period, and having a breeding range that is less that half the size of the winter range. However, the maximum daily consumption of 477 insects*km^-3^ of range is estimated to occur during fall migration when passage migrants are using a relatively small land mass, primarily in Central America (Table 4).

**Table 4:** Model based estimates of the biomass and number of insects consumed by Purple Martins during the breeding, migratory, and winter phases of its annual cycle.

Phase	Dates	Range Volume (million km^3^)	Biomass (kg*km^-3^)	Insects (km^-3^)	Insects (d^-1^*km^-3^)
Breeding	4/1 to 8/31	5.72	10.3 (3.0)	36,404 (10,066)	238 (66)
Fall Migration	9/1 to 9/30	2.97	4.0 (1.2)	14,313 (3,960)	477 (132)
Winter	10/1 to 2/28	11.60	3.9 (1.1)	13,713 (3,796)	91 (25)
Spring Migration	3/1 to 3/31	2.97	2.4 (0.7)	8,596 (2,377)	268 (74)

## Discussion

We estimated that Purple Martins consume a minimum of 262 billion insects annually, which is likely to be a significant component of aerial trophic systems in many regions. In the USA and Canada, where Purple Martins breed, they consume more than twice as much energy and insects * km^-3^ each year as they do elsewhere in their range. This is the first quantitative estimate of the magnitude of trophic ecology occurring in the aerosphere for this large and abundant aerial insectivore. As with any model, the accuracy of the analysis depends on the spatial and temporal accuracy of the underlying map and model parameters with respect to the distribution of birds within their range. Nonetheless, the modeling approach we developed here could be applied to other aerial insectivores to derive aggregate trophic impacts of this foraging guild at a variety of temporal and spatial scales.

There are a number of ecological and conservation related applications of the results of this model. For example, these estimates factor into the evaluation of potential direct and indirect ecosystem services that aerial insectivores provide for humans [28]. Purple Martins provide enjoyment to thousands of people who maintain their nesting structures and are avid supporters of their conservation. These birds also undoubtedly provide some benefit related to consumption of insect pests, although not mosquitos [23]. It is also possible that Purple Martins create significant ecosystem costs through trophic cascades. For example, Purple Martin’s consume predatory insects such as Odonates, which have been proposed for use in biological control programs because they consume insect pests and disease vectors that contribute to human health problems (e.g., [29]). Such complex trophic pathways add to the difficulty of quantifying the economic impacts of predatory habits [30]. Because of these complexities, accurate determination of economic value arising from ecological interactions remains beyond the scope of available data in most systems [9]. This type of analysis would, at least, require data on the insects being consumed, their impacts on the human economy, and the values of the people being impacted. The models we describe provide some estimates needed for this type of analysis of ecosystem services provided by aerial insectivores. However, data on some required elements do not currently exist. Future studies that work to gather these data and estimate ecosystem services from aerial vertebrate consumers in agricultural and other landscape types would be valuable [9].

It is also critical to recognize that monetary valuation is only one of several justifications for the conservation of aerial insectivores. Like all biodiversity, aerial insectivores, and the ecological processes in which they engage, have intrinsic value separate and apart from human valuations [31]. The Purple Martin is one member of a guild of aerial insectivores that includes many other North American bird (e.g., nightjars, swifts and swallows) and most temperate bat species; some of which are among the most common animals in North America. Many members of this guild are facing conservation concerns specific to particular habitats or regions. For example Boyles et al. [9] point to concern over pesticides, wind power, and white nose syndrome for bats, and among swifts and swallows, intensified agriculture and the resulting degradation of breeding habitat is a rising concern [32]. As the aerosphere becomes more crowded with devices used for energy development, communication, transportation, and remote sensing, the scale of human wildlife conflicts in this portion of the biosphere is likely to increase and the potential for large scale ecological and anthropological disturbances to exacerbate declines in aerial insectivores is readily apparent [7]. Accumulation of these impacts is a challenge for species conservation and maintenance of biodiversity of the aerosphere. The model analysis we report is a first step for understanding the magnitude of the trophic dynamics in the aerosphere and it points to a need for a more quantitative understanding of the ecology of aerial insectivores and their prey. A clear next step will be a spatially-explicit analysis of the distribution of the trophic impact of martins during the breeding season, a time period when maps with better spatial resolution are available.
